# Adnate Leaf-Base and the Origin of Ribs in Succulent Stems of *Euphorbia* L.

**DOI:** 10.3390/plants11081076

**Published:** 2022-04-15

**Authors:** Gustavo Arévalo-Rodrigues, Fernanda Hurbath, Erika Prado, Isabella Galvão, Inês Cordeiro, Diego Demarco

**Affiliations:** 1 Núcleo de Conservação da Biodiversidade, Instituto de Pesquisas Ambientais-PEFI, Sao Paulo 04301-902, SP, Brazil; arevalogustavo92@gmail.com (G.A.-R.); inescordeiro@sp.gov.br (I.C.); 2 Unidade Passos, Universidade do Estado de Minas Gerais, Passos 37900-106, MG, Brazil; fhurbath@gmail.com; 3 Departamento de Botânica, Instituto de Biociências, Universidade de São Paulo, Sao Paulo 05508-090, SP, Brazil; erikapradomaximo@usp.br (E.P.); isabellagalvao97@gmail.com (I.G.)

**Keywords:** plant development, vasculature, leaf traces, structure, microtomography, Euphorbiaceae

## Abstract

Stem succulence evolved independently in many plant lineages as an adaptation to arid environments. One of the most interesting cases is the convergence between Cactaceae and *Euphorbia*, which have anatomical adaptations mostly to increase photosynthetic capability and water storage. Our goal was to describe the shoot development in two succulent species of *Euphorbia* using light microscopy coupled with high-resolution X-ray-computed tomography. Collateral cortical bundles were observed associated with the stem ribs in both species. The analysis of vasculature demonstrated that these bundles are, in fact, leaf traces that run axially along a portion of the internode. That structural pattern is due to an ontogenetic alteration. During shoot development, the leaf-bases remain adnate to the stem near the SAM, forming an axial component. When the internode elongates, the leaf bundles stretch as cortical bundles. The meristematic activity associated with the bundles forms the stem ribs, as leaf veins near the node, and induce rib formation along the entire internode even in the portion where the leaf traces join the stele. In addition, heterochronic shifts are also involved in the evolution of the shoot system in these *Euphorbia*, being related to early deciduous reduced leaves and the transference of the main photosynthetic function to the stem. This study demonstrates for the first time the influence of leaf developmental shifts and stem rib formation in *Euphorbia* and sheds new light on the evolution of stem succulence.

## 1. Introduction

Among the numerous adaptive strategies found in arid environments, stem succulence plays a pivotal role in plant survival. This evolutionary innovation probably occurred in more than 30 families [1,2,3,4,5], as an important convergence which favored the occupation of environments lacking water by distinct angiosperm lineages [6]. This strategy involves organ thickening by means of the expansion of parenchyma (chlorophyll and water or starch storage tissues) in the cortex or pith [6,7,8,9]. A most remarkable convergence case is observed comparing cactiform species of *Euphorbia* (Euphorbiaceae) and cacti (Cactaceae) [10,11,12,13,14], but it is not restricted to these two families. Stem succulence based on parenchyma expansion has also been reported for several unrelated genera, such as *Caralluma*, *Duvalia*, *Echidnopsis*, *Hoodia*, *Huernia*, *Larryleachia*, *Pachypodium*, *Plumeria*, *Pseudolithos,* and *Stapelia* in Apocynaceae, *Othonna* and *Senecio* in Asteraceae, *Tylecodon* in Crassulaceae, *Pelargonium* and *Sarcocaulon* in Geraniaceae, *Dorstenia* in Moraceae, and *Cissus* in Vitaceae [1,2,4,5,9,15,16,17].

Despite the fact that many valuable data on the origin and structure of the succulence are available for leaves of some families [18,19,20] and stems of Cactaceae [9,21,22,23,24], little is known about the succulent stems of *Euphorbia* (Euphorbiaceae). Even though some xeromorphic characters related to succulence are apparently of the same morphological nature, their development and evolutionary shifts are practically unexplored, particularly in groups with non-succulent ancestors [14], such as *Euphorbia* [25]. The need for further studies to determine the origin of succulence in different lineages is reinforced by a study of Mauseth [9], who reported the lack of many features typically considered xeromorphic in succulent stems of 28 species from seven families other than Cactaceae.

*Euphorbia* L. is the largest genus of Euphorbiaceae and one of the largest within angiosperms [26], with about 2000 species occurring worldwide, especially in arid and semi-arid environments in the Tropics [27]. The genus has many life forms, such as herbs, geophytes, trees and shrubs, which encompass the major diversity of succulent species of the genus and are morphologically characterized as xerophytic cactiform species and pencil-stem species [25]. The cactiform species usually have organs modified into thorns and succulent and/or ribbed stems which are photosynthetic, at least in the young parts. The pencil-stem species have similar features, but the stems are thinner and permanently photosynthetic along the entire shoot system [25]. Remarkably, species of *Euphorbia* with those morphologies usually have highly reduced leaves but some other species retained large foliage leaves, decreasing the dependence on the stem photosynthesis, a trait much rarer in Cactaceae [25,28,29].

The subgenus *Euphorbia* is recognized by the most intricate evolution of xeromorphic growth form with five independent origins in the genus [25] and a wide diversity of species found in Africa and Madagascar. Fifteen species occur in the Neotropics [30], whose main monophyletic section is *Euphorbia* sect. *Brasilienses* V.W. Steinm. & Dorsey with five succulent pencil-stem species: *E. attastoma* var. *attastoma* Rizzini, *E. attastoma* var. *xanthochlora* Rizzini, *E. holochlorina* Rizzini, *E. phosphorea* Mart., *E. sipolisii* N.E.Br. and *E. tetrangularis* Hurbath & Cordeiro. These five species are shrubs, usually ramified, with succulent photosynthetic stems with 4–8 ribs, bearing reduced, early deciduous leaves. They are endemic to Brazil, occurring in environments with sandy soils such as “caatinga” (shrubland vegetation common to the arid climate of northeast Brazil) and rocky uplands [31].

Succulent stems usually have abundant water-storage tissue and chlorenchyma [6,7,8,32,33,34,35] and may have vascular modifications since the succulence is recurrently associated with anatomical changes in the vascular tissues [19,20,36,37,38,39,40]. Although the xeromorphic features of the cactiform *Euphorbia* are widely known, anatomical investigations of their stems and early deciduous leaves are lacking.

Among the few anatomical studies performed in the genus, the presence of cortical vascular bundles stands out as one of the striking unexpected features found in some species [9,30,32,41,42], also reported for some Cactaceae [9,43,44,45,46,47]. The main function assigned to these bundles is to improve the transport of photo-assimilates from the chlorenchyma to secondary vascular system and to provide mechanical support to the shoot [30,46,48]. Additionally, Solereder [32] and Metcalfe & Chalk [33] reported a high development of the cortex in succulent stems, mainly due to the activity of marginal meristem. However, its action mechanism remains unclear.

Our study aimed to investigate the development and vascular architecture of pencil stems and reduced leaves in *Euphorbia* in order to provide a model to understand the origin of some xeromorphic features in succulent stems of the genus.

## 2. Results

**Figure 1:**
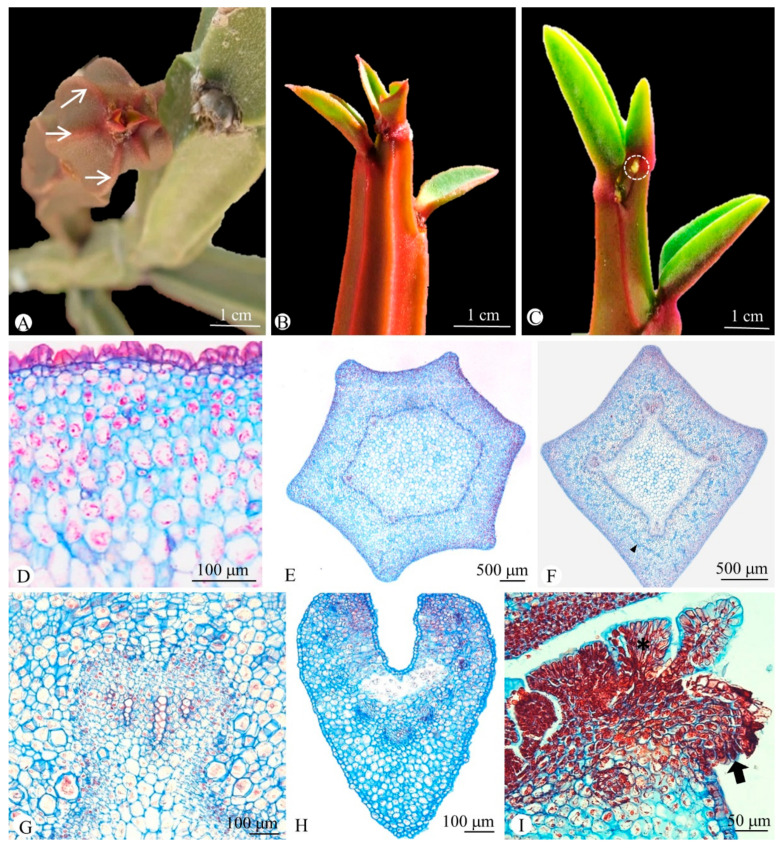
Stem morphology and anatomy of *Euphorbia attastoma* (**A**,**B**,**D**,**E**) and *E. tetrangularis* (**C**,**F**–**I**). Cross sections (**D**,**E**,**F**–**I**). (**A**) Stem ribs in frontal view (arrows) with leaves restricted to the shoot apex. (**B**,**C**) Note the spiral leaves in (**B**) and alternate, distichous leaves with stipules in the leaf base (dashed circle) in (**C**). (**D**) Detail of a rib showing many layers of chlorenchyma and a papillate epidermis. (**E**,**F**) Stem with a prominent vascular bundle in the eustele in each radius of the ribs, numbering six in *E. attastoma* (**E**) and four in *E. tetrangularis* (**F**). Note the numerous branched laticifers (arrowhead in (**F**)). (**G**) Detail of a vascular bundle of the eustele. (**H**) Median portion of the leaf with chlorenchyma and vascular bundles. (**I**) Detail of the leaf axil with colleters (asterisk) and one of the glomeriform stipules (arrow).

*Euphorbia attastoma* and *E. tetrangularis* are candelabriform shrubs with succulent branches of pencil-stem type. The leaves are reduced and restricted to the apex with early abscission (Figure 1A). Both species have ribbed stems, showing six ribs and spiral phyllotaxis in *E. attastoma* (Figure 1B) and four ribs and alternate, distichous leaves in *E. tetrangularis* (Figure 1C). The ribs are found only in the internodes, arranged in alternate groups of three projections, which coincide with the base of the leaves, which are simple and sessile.

### 2.1. Anatomy

**Figure 2:**
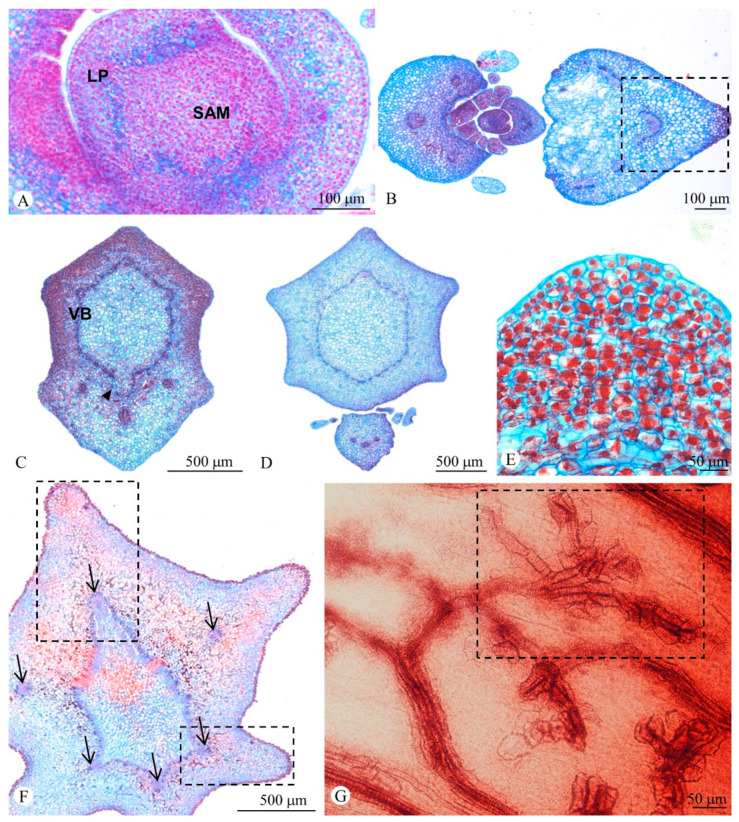
Ontogeny and vasculature of the shoot in *Euphorbia attastoma* (**A**,**C**,**D**,**F**) and *E. tetrangularis* (**B**,**E**,**G**). (**A**) Origin of the leaf primordium (LP) in the flank of the shoot apical meristem (SAM). (**B**) Leaves from distinct nodes in different developmental stages. Note the parenchymatic expansion of the midrib of the leaf (dashed square). (**C**) Nodal region showing three leaf traces associated with their respective ribs and the leaf gap (arrowhead). (**D**) Reduced leaf with three bundles and the ribbed stem. (**E**) Detail of the anticlinal and periclinal division of the ground meristem cells during rib development. (**F**) Divergence of leaf traces from the eustele, evidencing their relationship with the stem ribs (arrows). Note the profuse multiplication of parenchyma in front of each trace, forming the ribs (dashed squares). (**G**) Leaf terminal venation with areoles composed of tracheids (dashed square). VB = vascular bundle.

The stem of both species has a uniseriate papillate epidermis with stomata and a cortex particularly thicker in the ribbed region due to the presence of dozens of layers of parenchyma (Figure 1D). The cortex is divided in two regions: an outer cortex formed by chlorenchyma with elongate cells near the epidermis and an inner cortex containing several layers of parenchyma with starch grains. The vascular system is arranged in an eustele of irregular shape (polygonal), whose angles vary according to the number of ribs (Figure 1E,F). Each angle has a large bundle, which is considerably larger than the other bundles (Figure 1G). In addition to the stele, vascular bundles are observed in the cortex, opposite to the ribs, whose number varies according to the number of ribs (Figure 2A–F). Branched laticifers occur throughout parenchyma and vascular bundles (Figure 1F).

The leaves are relatively small, varying from 6.7–11 × 2.2–3 mm and have a uniseriate epidermis with stomata and chlorenchyma (Figure 1H). They have three vascular bundles in the base, which subsequently branch in five to nine in the median portion (Figure 1H). Laterally to the base of the leaf blade, a pair of glanduliform stipules with papillate epidermis are formed together with a profusion of sessile colleters, which extend from one stipule to the other along the leaf axil (Figure 2B). These colleters are formed by a secretory palisade epidermis and a non-secretory parenchyma core (Figure 1I).

### 2.2. Shoot Ontogeny and Vasculature

The leaf primordium originates from the peripheral zone in the flank of shoot apical meristem (SAM). The initiation occurs in regular plastochrons, which vary according to the phyllotaxis (Figure 2A). During the expansion of the leaf primordium, its base remains united to the stem primordium, becoming part of the axis. The subsequent intercalary growth of the stem primordium elongates this adnate region, and the leaf-base is stretched along a portion of the internode, where the leaf traces are observed as stem collateral cortical bundles.

**Figure 3:**
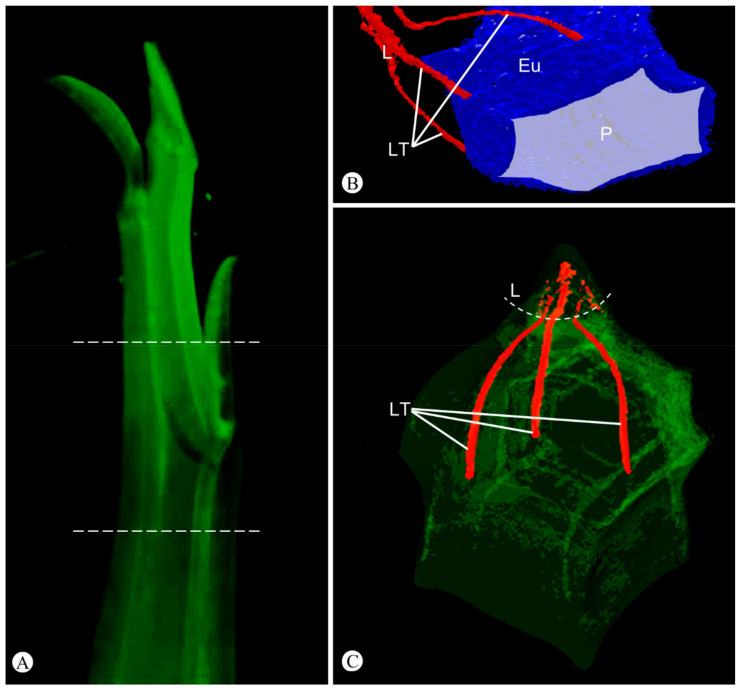
Microtomography with 3D-reconstruction of the shoot of *Euphorbia attastoma*. (**A**) General view of the shoot. (**B**,**C**) Reconstructions of the area enclosed by the dashed lines in (**A**). (**B**) Detail of the leaf traces (LT in red) connected to the eustele (Eu in blue) surrounding the medullary parenchyma (P in gray). Note that the three leaf traces do not branch until reach the free portion of the leaf (L). (**C**) Nodal region showing the leaf traces (LT in red).

In this second stage of the shoot morphogenesis, the ground meristem in front of the collateral cortical bundles (leaf bundles) generates numerous layers of parenchyma (Figure 2E), which correspond to leaf veins. Ontogenetically, these veins are the ribs of the stem, formed by the cortical bundle and a large amount of parenchyma, which doubles the thickness of the cortex. In regions without ribs, the cortex of both species is 1.5–2 mm thick, but in the rib radius the cortex is about 4–4.5 mm. This relationship of the leaf base as an axial component can also be perceived through the analysis of vasculature (Figure 2 and Figure 3), but it is not restricted to the portion of leaf-base adnation. In fact, since the leaf-base becomes an axial component in the shoot apex, its incorporated vasculature induces the proliferation of the ground meristem basipetally, both in the region with cortical bundles and in the region without bundles, which, actually, corresponds to the most length of the internode.

Leaf morphogenesis is rapid. The free portion of the leaf, found around 1.5–2 cm above the node, is comprised exclusively of the leaf blade. Early in leaf development, meristems differentiate into mature tissues, stopping the leaf expansion. As a consequence, the leaves remain small and are also early deciduous, being restricted to the apex portion of the shoot. Thus, the photosynthesis of the plant is supplied almost entirely by the stem with adnate leaf-bases.

Shoot vasculature varies according to evolutionary developmental shifts. In the nodal region, three leaf traces diverge from the stele and occupy a peripheral position in the cortex in the region of the stem rib, running parallel to the surface along the internode, profoundly altering the stem morphology. These traces remain unbranched until the base of the free portion of the leaf (Figure 3, Supplementary Material Video S1). In the leaf blade, the three bundles ramify, forming secondary and tertiary veins, which terminate with tracheids in the areoles (Figure 2G).

## 3. Discussion

The present study revealed the origin and nature of the ribs of the succulent stems in two Neotropical species of *Euphorbia* for the first time. Using developmental analysis and 3D-reconstruction techniques, we were able to demonstrate that the leaf-base is congenitally adnate to the stem, as an axial component, and the development of the three main veins of this base induces rib formation along the entire internode of the succulent pencil stems.

Succulent stems are common in *Euphorbia*, exhibiting ribs with reduced, early deciduous leaves. Rudimentary leaves occur in other New World lineages with ribbed stems of the subgenus *Euphorbia*, such as in *E. pteroneura* A. Berger (sect. *Euphorbiastrum* (Klotzsch & Garcke) Boiss.), a Mexican species (sensu Dorsey et al. [27]). Ribbed stems also occur in herbs, particularly in *E*. sect. *Stachydium* Boiss., the sister group of *E*. sect. *Brasilienses*, e.g., *E*. *heterodoxa* Müll. Arg., an endemic species from rocky outcrops in Northeastern of Brazil. Other succulent lineages within the genus also have rudimentary leaves with succulent ribbed and/or tuberculate stems. Those species belong to the Old World and are currently classified in the subgenus *Euphorbia* and *E*. subg. *Athymalus* Neck. ex Rchb. [27,49]. Given the diversity of species bearing ribbed stems in unrelated lineages, the main common trait is the presence of rudimentary leaves. This fact is certainly involved in the stem organogenesis in *Euphorbia* since the stem ribs are concomitantly produced with the leaf primordium, exhibiting an easily recognizable relationship between both organs.

Restriction of leaf blade development and transference of function to stem, which becomes the main photosynthetic organ of the plant, is usually related to succulent stems in arid environments [9,48,50,51,52,53]. The enlargement of the cortex of these stems appears to be related to the expansion of the photosynthetic tissue, in addition to the increment in water and starch storage [9,48,51,54,55,56,57,58].

The cortex may be extremely broad in Cactaceae due to cortical bundles which produce intrafascicular secondary phloem and xylem in many species [9,48,50]. On the other hand, we did not observe any expansion of the stem tissues in *Euphorbia* due to vascular proliferation of the cortical bundles. Actually, the non-separation of the leaf-base from the SAM flank during the leaf primordium development seems to be the reason for the succulence of the pencil stem in the genus. The maintenance of the leaf-base forming an axial component has already been reported for plants of other families [59].

Fusion of the leaf adaxial side to the stem is not an uncommon process for bud protection in xerophytes since it reduces the water loss to the environment. This process usually involves expansion of the leaf-base. In succulent species of Aizoaceae and Amaranthaceae, a large expansion of the leaf-base around the SAM and a reduction in the leaf blade have previously been reported [18,20,60]. This foliar characteristic appears to have evolved multiple times in these two families, coinciding with the aridification of southern Africa in the Late Miocene [60,61,62]. However, there is no fusion of the leaf-base to the stem, as observed in *Euphorbia*. Conversely, leaf fusion may be total in other lineages. In Podostemaceae, the leaf-base is completely united to the promeristem, originating shoots apparently devoid of SAM in the subfamily Podostemoideae [63].

In general, main xeromorphic features of the succulent stems are the increase in parenchyma tissue of the cortex or pith, reduction of leaf size and number, and establishment of mechanisms for water protection and its storage [9]. Regardless of the origin of expanded cortical tissues of the succulent stems, the enlargement is associated with the presence of cortical bundles. The emergence of cortical bundles was a key innovation in the evolution of Cactoideae (Cactaceae), which allow some species to develop cortices up to 30 cm thick keeping all their tissues hydrated [48,64]. If the cortex is unvascularized, the slow transport of water by diffusion appears to limit the increase in cortex thickness [55,64].

Extrastelar vascular bundles, such as the collateral cortical bundles (leaf traces) observed in the Neotropical *Euphorbia* sect. *Brasilienses*, have also been reported for *E. weberbaueri* Mansf. (*E*. sect. *Euphorbiastrum*) (Klotzsch & Garcke) Boiss. [30], another Neotropical species with ribs very similar to those described herein, and also for African species as *Euphorbia fortuita* A.C. White, R.A. Dyer & B. Sloane, *Euphorbia horrida* Boiss., *Euphorbia obesa* Hook. F. and *Euphorbia officinarum* L. [9]. Those additional bundles have been reported for 55 eudicot families, extending along the internodes, as cortical bundles or medullary bundles [33]. In the case of cortical bundles, Howard [65] observed that their relationship with the leaves varies depending on the group analyzed and can be found in the stem (1) unrelated to the leaf vasculature, (2) partially related to leaf vasculature or (3) completely related to leaf vasculature. This third condition is the one discovered here in *Euphorbia attastoma* and *E. tetrangularis*. This variation indicates that the mere occurrence of cortical bundles in the stem does not necessarily imply adnation of the leaf to the stem. The main example is the cacti, whose extensive set of cortical bundles, observed in addition to the leaf/bud traces, are truly cauline, derived from procambial cells originated in the cortical area near the shoot apical meristem [44,48,64].

Despite the fact that the origin of ribbed stems has not been investigated in other species of *Euphorbia*, further anatomical studies may reveal similarities in relation to our results, indicating the likely evolution of this character in other clades of the genus. Mauseth [9] found that the leaf traces are related to the stem ribs in nine species of *Euphorbia*. If this relationship exists between this type of stem succulence and leaf traces in other species, we can assume that the number of ribs and their disposition depends on the phyllotaxis and plastochron. This hypothesis is supported by the difference observed between *E. attastoma* (six ribs) and *E. tetrangularis* (four ribs). This is related to the three leaf traces that diverge from the eustele in different times during development, indicating a case of heterochrony in closely phylogenetically related species. The ribs are surely associated with the stem thickness, allowing the candelabriform morphology observed in *E*. sect. *Brasilienses* and seems to be important to accommodate seasonal expansion and contraction of stem [66].

**Figure 4:**
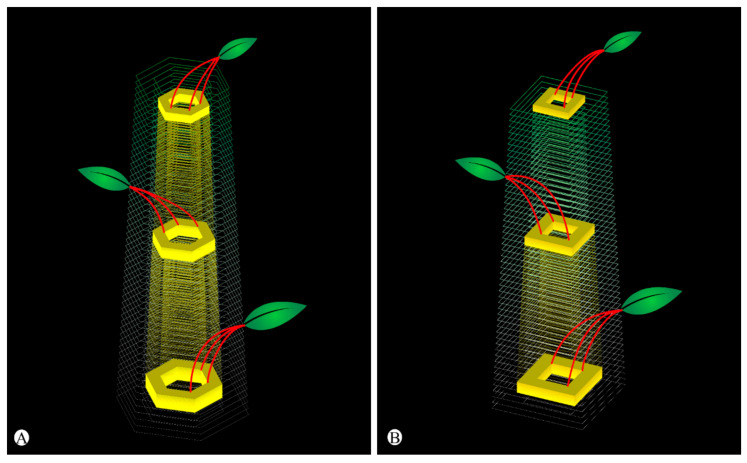
Diagrams showing the leaf traces diverging from stele in *Euphorbia attastoma* (**A**) and *E. tetrangularis* (**B**). Note that the leaf traces (red) diverge in groups of three in both species.

The evolutionarily shift of plants from one form into another involves modifications of the developmental patterns [48]. In this study, we verified that during the initial development of leaf primordia, the leaf-base remains adnate to the developing stem in *Euphorbia*. This congenital fusion of tissues displaces the orientation of the leaf traces, which are oblique/horizontal and run directly to the leaf-base at the nodes in the vast majority of angiosperms [65], to run roughly parallel to the surface of the stem along a short portion of the internode (Figure 4).

**Figure 5:**
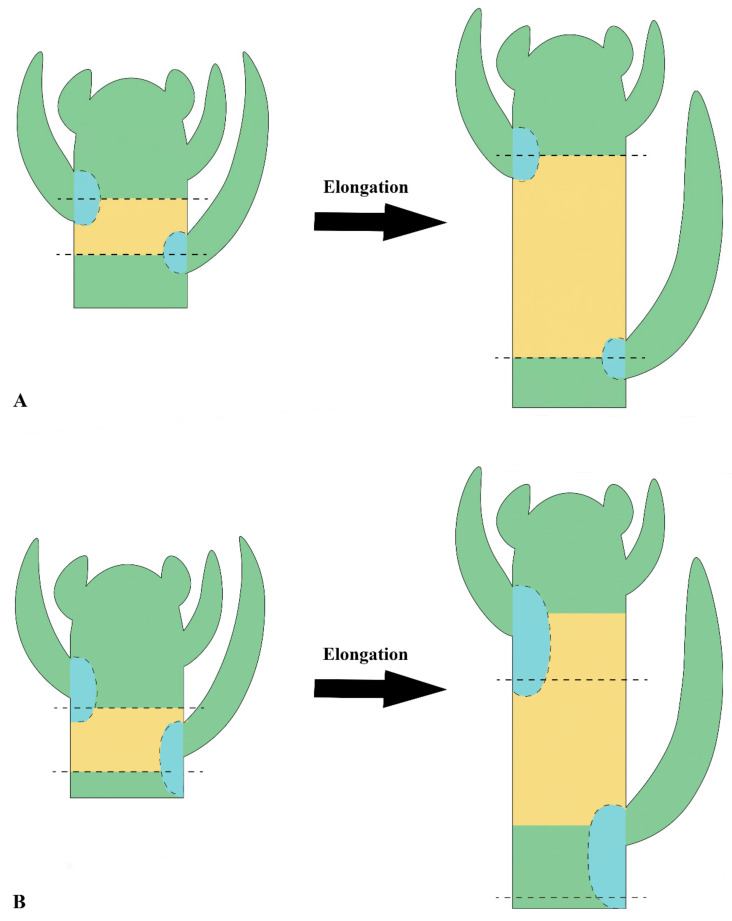
Schematic drawings of two stages of shoot development. (**A**) Regular shoot development. Note that the leaf-base participation in the axis is restricted to its point of origin at the node, even after the internode elongation. (**B**) Shoot of *Euphorbia attastoma* and *E. tetrangularis*. Note that adnate leaf-base forms an axial component, displacing the leaf to a region above the node. The fused portion elongates along with the internode. The origins of axial tissues were colored in one plastochron. Dashed line: node; blue: leaf-base component; yellow: cauline component; green: indiscriminant components.

Fusion of the leaf-base to the stem is not an uncommon event in angiosperms [67], but the developmental process observed in *Euphorbia* appears to be an evolutionary novelty and is described for the first time in this study. Despite the fact that the general leaf development is well known and widely analyzed in textbooks and articles, the origin of the stem is often neglected. After the formation of the leaf primordia in the shoot apex and the establishment of the nodal regions, there is an elongation of the internode by multiplication and elongation of the cells of the primary meristems [65]. This second stage of the stem morphogenesis has been altered in the *Euphorbia* pencil stem. When the internode elongates, the leaf-base united to the stem also elongates (Figure 5), stretching the leaf traces along the internode. Consequently, the leaf is “displaced” to another region above its point of origin (Figure 4).

The increment of foliar tissues into the stem considerably expanded the cortex, increasing its photosynthetic capacity (Figure 5). Additionally, there is an early maturation of the leaf meristems, interrupting the leaf blade development and producing very reduced leaves. This type of developmental shift of the tissue differentiation timing is known as heterochrony and has been shown to be one of the main processes in plant evolution [68]. In addition, some heterochronic changes may lead to transfer of function (heterotopy) due to spatial relationships that change over time [69]. This can be observed in the cactiform species of *Euphorbia*, whose stem becomes the main photosynthetic organ of the plant due to the early leaf abscission.

Despite our observation that cortical bundles (leaf traces) elongate for a short region of the stem above the node, these vascular bundles undoubtedly stimulate parenchyma proliferation, forming the ribs continuously along the entire internode. To date, it is not possible to infer which endogenous signal is responsible for this change in stem tissues. However, previous studies show that the hormonal stimulus responsible for the differentiation of procambium, and consequently the vascular bundles in shoots, is the auxin. Polar auxin gradients induce procambium differentiation along the path of its flow and may regulate vascular adaptation to the plant’s environment [70,71]. Thus, if auxin is related to the differentiation of cortical bundles, we can hypothesize that auxin is also related to rib differentiation regardless of the presence of cortical bundles from one node to the other, deviating from the expected transport pathway due to the adnation of the leaf base to the stem. Further immunocytochemical studies are needed to definitively verify this hypothesis and clarify the physiological regulation of succulence evolution in *Euphorbia*.

## 4. Materials and Methods

For this study two species of *E.* sect. *Brasilienses* were selected: *E. attastoma* (Hurbath 853, 854) and *E. tetrangularis* (Hurbath 844). The samples were collected from three individuals of each species cultivated in glasshouse at Instituto de Biociências at the Universidade de São Paulo. Vouchers of the species were deposited at SP Herbarium.

Shoots were fixed in FAA for 24 h (formalin, acetic acid, 50% ethanol 1:1:18 *v*:*v*) [72] and then stored in 70% ethanol. Entire leaves were first cleared using 100% ethanol, then treated with 10% sodium hydroxide for 2 h, followed by 5% sodium hypochlorite. The leaves were stained with 1% safranin and mounted in Kaiser’s glycerin gelatin [73]. For anatomical analyses, shoot apices were isolated, dehydrated in a butyl series [72], embedded in Paraplast^®^ (Leica Microsystems, Wetzlar, Germany) and transversely or longitudinally sectioned using a Leica RM2145 rotary microtome. Sections 12 µm thick were stained with astra blue and safranin [74], and the slides were mounted in Permount^®^ (Fisher Scientific, Pittsburgh, PA, USA). The photomicrographs were taken using a Leica DMLB light microscope coupled with a digital camera.

For high-resolution X-ray-computed tomography (HRXCT), we used the fixed shoots of *E. attastoma*, which were treated with phosphotungstic acid in 70% ethanol for one week. Subsequently, the samples were dehydrated in an ascending ethyl series containing 1% phosphotungstic acid (1:1; *v*:*v*) and wrapped with parafilm in a tube filled with 100% ethanol. Finally, the samples were scanned using a SkyScan 1176 microtomograph (Bruker, Billerica, MA, USA). The exposure time was approximately 4 h per sample and for the 3D reconstruction we used the software CTVox and CTVol, 3D Doctor (Able Software Corp., Lexington, KY, USA) and 3D Slicer. Image sequences and the video (Supplementary Material) were segmented automatically and manually.

The vasculature diagrams were made based on the microscopic observations and the high-resolution X-ray-computed tomography, using the software Cinema 4D (Friedrichsdorf, Hesse, Germany) and Adobe Photoshop (San Jose, CA, USA).

## 5. Conclusions

We demonstrate for the first time that the succulence of the pencil stem in *Euphorbia* is due to the incorporation of the leaf-base as an axial component during shoot development. This joining of tissues doubles the thickness of the cortex of stem and its amount of photosynthetic tissue. The elongation of the internode joined to the leaf-base transforms the leaf traces into cortical bundles that are associated with proliferation of the parenchyma, forming the stem ribs. A likely hormonal signal from the leaf is transported basipetally along the entire internode, inducing rib formation regardless of the presence of cortical bundles. Heterochronic shifts are also involved in the evolution of the very small leaves of *Euphorbia*, their early abscission and the transference of the main photosynthetic function to the stem. A major sampling is needed to evaluate the specific role of these features in the adaptive success of the *Euphorbia* and its diversification in arid environments.

## Data Availability

All figures and tables of this manuscript have been unpublished and were made specifically for this article.

## Supplementary Materials

The following supporting information can be downloaded at: https://www.mdpi.com/article/10.3390/plants11081076/s1, Video S1: Microtomographic reconstruction of the shoot of *Euphorbia attastoma*.
